# Assessing a Metacognitive Account of Associative Memory Impairments in Temporal Lobe Epilepsy

**DOI:** 10.1155/2016/6746938

**Published:** 2016-09-19

**Authors:** Nathan A. Illman, Steven Kemp, Céline Souchay, Robin G. Morris, Chris J. A. Moulin

**Affiliations:** ^1^Institute of Psychological Sciences, University of Leeds, Leeds LS2 9JT, UK; ^2^Department of Clinical Psychology, St James' University Hospital, Leeds, UK; ^3^Laboratoire de Psychologie et Neurocognition (CNRS 5105), Université Grenoble Alpes, 38040 Grenoble, France; ^4^Department of Psychology, King's College London, Institute of Psychiatry, Psychology and Neuroscience, De Crespigny Park, London SE5 8AF, UK

## Abstract

Previous research has pointed to a deficit in associative recognition in temporal lobe epilepsy (TLE). Associative recognition tasks require discrimination between various combinations of words which have and have not been seen previously (such as old-old or old-new pairs). People with TLE tend to respond to rearranged old-old pairs as if they are “intact” old-old pairs, which has been interpreted as a failure to use a recollection strategy to overcome the familiarity of two recombined words into a new pairing. We examined this specific deficit in the context of metacognition, using postdecision confidence judgements at test. We expected that TLE patients would show inappropriate levels of confidence for associative recognition. Although TLE patients reported lower confidence levels in their responses overall, they were sensitive to the difficulty of varying pair types in their judgements and gave significantly higher confidence ratings for their correct answers. We conclude that a strategic deficit is not at play in the associative recognition of people with TLE, insofar as they are able to monitor the status of their memory system. This adds to a growing body of research suggesting that recollection is impaired in TLE, but not metacognition.

## 1. Introduction

Temporal lobe epilepsy (TLE) is typified by episodic memory impairment. In recent years the memory deficit has been increasingly researched in terms of the contribution of two arguably separable processes, that of recollection and that of familiarity. In recognition memory, recollection refers to the qualitative process which allows specific details of the study episode to be retrieved through associations between the test item and components of the study context, the other items that were studied, and the physical characteristics of the study item itself (e.g., size, shape, and colour). In the absence of intact recollection, then the parallel process of familiarity may aid memory; recognition based on familiarity is done in the absence of retrieving contextual or associative information but still provides a relatively good indicator of prior exposure through a feeling of “oldness.” A number of studies have now produced evidence for recollection as the locus for impairment in TLE, as established by exploring associative recognition memory [1–8].

This emphasis on recollection derives from the role of the medial temporal lobe (MTL) in specific recollection processes (see [9] for a review). However, TLE is also characterised by damage to wider reaching brain networks and impairments are found in domains other than memory alone, such as intelligence, language, working memory, and executive function [10, 11]. In particular, the MTL is part of a network which involves the frontal lobes and this frontal-temporal interaction may be involved in higher-order memory retrieval and decision making processes. Indeed, one recently tested idea is that some of these memory difficulties are, at least in part, metacognitive [12], that is, being related to higher-order strategic and reflective processing of memory mechanisms. Here we take a metacognitive approach to better understand the specific memory processes at fault in TLE by examining the strategic control of memory in relation to confidence judgements. In particular we are interested in how the characteristic associative deficit briefly described above is related to reports of subjective confidence. To introduce this idea, we first look in detail at the type of task that best displays the critical memory deficit found in TLE.

In a typical associative recognition task, a list of word pairs are first presented and recognition memory is subsequently tested by requiring participants to discriminate between different types of pairs; pairs presented previously are to be endorsed, whilst rearranged combinations of previously studied words are to be rejected. Hence, familiarity with initially presented words will not offer enough information to differentiate intact from rearranged pairs. Such tests have therefore been demonstrated to be a measure of recollective or relational memory and have been used extensively in studies of amnesia along with item recognition tests to compare recollective dependent processing with familiarity based memory [13–16]. This type of associative recognition is, however, not measured routinely in clinical neuropsychological assessment of TLE. Instead, impairments in arbitrary associations for verbal stimuli—often tested via cued-recall—are used as lateralising evidence for left TLE (LTLE) ([17]; see [18] for a critical discussion).

To our knowledge, only one study has specifically addressed the relative contribution of recollection and familiarity impairment to item and associative memory deficits in TLE (postoperative patients; Cohn et al. [19]). Drawing on their earlier programme of research [20, 21], the authors examined performance on two associative memory measures. First, associative identification involves endorsing unrearranged (intact) pairs and rejecting rearranged old, half-old, and new pairs. Cohn and colleagues suggest that this ability requires successful binding of information during encoding as well as the initiation of strategic retrieval operations during the test. The second measure is associative reinstatement, which represents the gain in item memory when word pairs are reinstated into their original encoding context (intact pairs), compared to their rearranged form. Cohn et al. [19–21] demonstrate that associative reinstatement is a relatively purer measure of relational binding ability dependent on the medial temporal lobe (MTL), relying less on frontally mediated strategic retrieval during the recognition test. Associative identification is derived from performance on an associative identification recognition task; item memory and associative reinstatement measures are derived from a separate pair recognition task in which participants discriminate old intact and rearranged pairs from new and half-old pairs. The instructions for responding in these two tasks mirror the process dissociation procedure (PDP) paradigm [22–24] because of the way recollection and familiarity processes are used. For example, the associative identification task is akin to a PDP exclusion condition as recollection is required to only endorse original studied pairings and reject any other permutations. The pair recognition task is akin to a PDP inclusion condition because familiarity alone is sufficient to accept rearranged or intact pairs, although recollection may still be used of course. Furthermore, hit rates and false positives (FPs) across tasks can be used to calculate estimates of recollection and familiarity (see [19]). In a group of postsurgical TLE patients with MTL resections, Cohn et al. [19] showed that associative identification, associative reinstatement, and recollection measures for both LTLE and right TLE (RTLE) patient groups were significantly below that of controls. They found familiarity estimates to be significantly reduced only in the LTLE group. Although a number of complex objective memory measures can be derived mathematically from the two recognition tasks in Cohn et al.'s study, the failure of TLE patients to reject rearranged word pairs in the associative identification task provides the most parsimonious and convincing evidence of an inability to use recollection to overcome familiarity of two previously seen items. To illustrate, the combined mean proportion of FPs to rearranged items for patients with left hemisphere language dominance or left/right language nondominance was 40%; in contrast, controls only falsely endorsed 16% of such items, on average. As a concrete example, consider the two studied word pairs,* holiday-flower* and* fortune-record*. When presented at test with the rearranged pair,* holiday-record*, healthy controls not only will experience familiarity for the two items but also will proceed to recollect at least one of the original pairings, allowing them to reject the item (i.e., recall-to-reject). TLE patients, on the other hand, are more likely to fail at the point where familiarity based recognition is successful; insufficient recall of the true formative association of either word results in incorrectly endorsing the pair as being “intact.” Note that a recall-to-accept strategy works in the same fashion for intact pairs.

There are several potential explanations for this memory error. The first, as suggested above, is that a recall-to-reject strategy has been unsuccessful due to a failure of recollection and the person is falsely seduced by the familiarity of the two items; they recognised each word but did not remember that each was from a different pair. We were motivated by a second (not unrelated) explanation which draws on recent conceptualisations of recollection as a fractionated process [9, 25]; it views this kind of memory error as a failure in* metacognition*. Recollection has been suggested to be comprised of a mnemonic element (i.e., contextual retrieval) and a metacognitive control component (i.e., responding appropriately at retrieval to that information held in mind). That is, people with TLE may fail to recall the requisite information since they act inappropriately in response to mnemonic cues due to the high subjective familiarity of the items and so fail to adopt the most beneficial “recall-to-reject” strategy. We might imagine that if a strategic error in the regulation of memory is behind the profile of performance in TLE, then participants will be overly confident of their recognition decision on such items.

Our argument is that the rejection of familiar items in rearranged pairs may in fact require strategic processing. In order to reject familiar items, one must be aware of the familiarity and act upon it; one must make a metacognitive decision to search memory to see whether the items are arranged correctly or not by the retrieval of material. Thus, either there is a lack of the correct material to base this decision upon (i.e., they cannot recollect the combination of items, despite trying), or perhaps they do not initiate the recollection search; they act on the familiarity but do not detect the need to retrieve more specific information from the time of encoding.

A direct test of this is to use a metacognitive task, whereby we can examine the subjective experience at the point of making this decision. Using confidence judgements, we imagine that if participants are aware of and try to recollect source information—the earlier encoded sentence—for familiar pairs, they will have a low confidence. If, on the other hand, they fail to detect the difficulty and thus fail to initiate the recollect-to-reject strategy, we might expect a high confidence rating (because the mere familiarity for the item will lead to high levels of confidence). In this way, we are using confidence judgements to explore whether, given the putative recollection deficit in TLE, participants are nonetheless aware of where they should be applying recollection to disambiguate familiar items in rearranged pairs. For a fuller account of metacognition in associate recognition tasks see Hines et al. [26].

Existing research into metacognition in TLE has been inconsistent, meaning further exploration of the link between memory and its subjective appraisal is of continued importance. Two early studies suggested that patients with TLE made inaccurate predictions of future memory performance [27, 28]. Notably, Prevey et al. [28] found that TLE patients overestimate their memory performance, in line with our hypothesis concerning overconfidence in recognition memory decision making. In contrast, a programme of research by Howard and colleagues [12, 29, 30] found no impairment in metacognitive monitoring in TLE patients, despite their lower overall memory ability. Of note, a further study by Fleming et al. [31] utilised a recognition memory paradigm with subjective confidence judgments, comparing postsurgical mesial temporal lesion patients and those with anterior prefrontal lesions. Their aim was to compare metacognitive accuracy across different domains in these patient groups and hence used a perceptual judgment task alongside the memory test. Like Howard et al.'s programme of research, these authors did not find any impairment in metacognition in either domain in the TLE group. However, the memory task in this study was a simple two-alternative forced choice procedure that is not adequate to investigate the fractionation of different MTL-related memory processes such as recollection and familiarity, as in an associative recognition paradigm.

In short, there have been divergent findings in the literature regarding metacognitive deficits in TLE and the methods used have not yet comprehensively assessed how awareness functions alongside specific MTL memory processes. Clinically, a well-recognised phenomenon in this patient group is the lack of correspondence between subjective memory estimates and objective performance (see [32] for a review); whilst this area of research has been well covered, experimental studies are important because they are able to contribute to the understanding of the dynamics between memory performance and how one perceives their actual memory.

Thus, in this study we explore the issue of the impact of metacognition on recollection and familiarity judgements measured using an associative recognition memory test in TLE. If metacognition is indeed intact in this group, we hypothesised this to be expressed as a lower level of confidence for this difficult task. Nonetheless, we should see a preservation in the accuracy of confidence: confidence should be significantly lower for incorrect than correct responses in the TLE group. This second part of the hypothesis is critical; if we found only a main effect of confidence between patients and controls, it may be due to levels of expectation or prior beliefs about memory function. The sensitivity to the different types of pairs and whether or not the response is correct are necessary to demonstrate metacognitive competence.

To sum up, we compared recognition confidence between stimuli types that relied more, or less, on recollection and familiarity. For example, correct recognition of intact pairs in both tasks is reliant on the contribution of recollection and familiarity, whilst, in the associative identification task, correct responses to rearranged items require the more strategic recall-to-reject recollective like process. In the pair task, familiarity alone is sufficient for correct recognition of these. Although rearranged pairs seemed critical to test a metacognitive account, a more general interest was in patients'* sensitivity* to task demands and how* accurate* their confidence was, that is, whether their confidence would show a normal pattern whereby incorrect responses are assigned lower confidence than correct responses.

## 2. Method

### 2.1. Participants

Twenty-two patients with TLE (M = 41.32 years; SD = 11.22; range = 18–57) and 18 controls (M = 37.72 years; SD = 13.93; range = 24–58) participated in the study. TLE patients either were recruited directly from neurology/clinical psychology outpatient clinics at Leeds Teaching Hospital NHS Trust sites (*N* = 11) or volunteered in response to an advertisement in a bimonthly publication produced by Epilepsy Action charity (*N* = 11). The control group was comprised primarily of spouses, partners, or friends of the TLE participants. Neither patients nor controls received compensation for participation. TLE participants all had diagnosis confirmed by an epilepsy specialist neurologist, confirmed by clinical data, for example, including EEG, MRI/CT, and semiology. Both groups met the following criteria: (1) aging between 18 and 65 years; (2) English being their native language; (3) being free from neurological (other than epilepsy in the patients) or psychiatric condition; and (4) having full scale IQ > 75. All participants provided written consent before proceeding with the study. NHS ethical approval was granted from Leeds Central Research Ethics Committee and by the institutional review board from the Institute of Psychological Sciences, University of Leeds.

#### 2.1.1. Demographic Characteristics

A summary of demographic characteristics for the two groups and clinical features for the TLE group are presented in Table 1. One-way ANOVA revealed no significant difference between groups in terms of age, years of formal education, and the National Adult Reading Test (NART; [33]). Wechsler Adult Intelligence Scale (WAIS) predicted full scale IQ (FSIQ) (*F*s < 1). A Chi-square test revealed proportionately more women in the TLE group, *χ* ^2^(1) = 6.08, *p* = 0.01.

Nine TLE patients had left epileptic foci, two who had undergone resective surgery. Nine TLE patients had right foci, three underwent surgery, and a further four had bilateral foci. Epilepsy surgery ranged from 5 to 11 years prior to date of testing. Noting the heterogeneous nature of the patient sample, the aim of the present study was not to provide an empirical understanding of memory and metacognition with respect to laterality or lesional status in TLE (however, see Table S3 in Supplementary Material for further outline of patient clinical characteristics, available online at http://dx.doi.org/10.1155/2016/6746938). The sample was suitable for our aim of testing the hypothesis that the deficit in associative recognition would manifest itself as an inaccurate assessment of the confidence for the critical item pairs. For this reason and due to the small number of patients with LTLE, RTLE, and bilateral TLE (BTLE), we focus all subsequent analyses on the epilepsy group as a whole.

#### 2.1.2. Neuropsychological Assessment

Tests were selected such that IQ, memory, and executive function were assessed in patients and controls. Reading ability and IQ were tested via the National Adult Reading Test (NART; [33]) and Wechsler Abbreviated Scale of Intelligence (WASI; [34]). Immediate visual (faces) and verbal recognition memory was assessed by the Warrington Recognition Memory Test (WRMT; [36]). Visual and verbal immediate and delayed recall were assessed with subtests from the BIRT Memory and Information Processing Battery (BMIPB; [35]), a robust UK-normed test battery. This included complex figure recall, story recall, and list learning in which the sum of five recall trials was used as a measure of learning, followed by auditory presentation of a second (interfering) list and ending with a postinterference recall trial. Working memory was assessed using Wechsler Memory Scale-third edition (WMS-III) digit span forwards and backwards [39] and executive retrieval and language function were measured via verbal and category fluency tasks from the Delis-Kaplan Executive Function Scale (DKEFS; [37]).

#### 2.1.3. Neuropsychological Data

See Table 2 for neuropsychological data and one-way ANOVA tests between patients and controls on all measures. For clinical reasons, two patients completed a slightly different battery of memory and intelligence tests. One patient completed the Wechsler Adult Intelligence Scale-IV (WAIS-IV [40]) and Wechsler Memory Scale-IV (WMS-IV [41]); her scores were in the average range for intelligence (WAIS-IV FSIQ = 111) and within normal limits for auditory (*z* = −0.47) and verbal (*z* = 0.20) memory indices of the WMS-IV. The other patient completed the WAIS-IV instead of the WASI and gained a FSIQ of 104. (One further TLE patient also dropped out of the study before any neuropsychological assessment could be completed—in this case and for the between-groups analysis of IQ scores, these participants were given prorated scores based on the group mean.)

One-way ANOVAs indicated that there is no difference between patients and controls in WASI verbal IQ, performance IQ, and FSIQ. Thus, our patient and control group were adequately matched in terms of premorbid and current intellectual function.

For memory and executive function, significant differences (with patients performing worse than controls) were found in the following measures: BMIPB immediate figure recall, delayed figure recall, postinterference list recall, immediate story recall, delayed story recall, WRMT words, and WRMT faces.

The tests that did not yield a significant difference included the following: BMIPB list learning, FAS verbal fluency, category fluency, digit span forwards, and digit span backwards. Thus, the present TLE patient group displayed a pattern of impaired performance on a range of anterograde verbal and visual memory tests compared to controls, whilst measures of executive function and working memory remained relatively intact.

Hospital anxiety and depression scale (HAD scale [38]) depression levels did not differ significantly between patients and controls. The TLE group had slightly higher levels on the anxiety scale, but the mean level falls outside the suggested clinical cut-off [42].

### 2.2. Materials

The experiment was based on the procedure used by Cohn et al. [19]. Two sets of 96 words were created and then each set was divided into 8 lists of 12 word pairs. For each word pair, semantically unrelated nouns were used, with seven-letter words for the first item in the pair and six for the second item in the pair. Word frequency was obtained using the SUBTlex corpus and imageability values were obtained from the MRC psycholinguistic database [43]. Frequency values ranged from 0.22 to 240.94 words per million, with a mean value of 26.26 for the seven-letter words (SD = 44.34) and 27.06 (SD = 35.14) for six-letter words. Mean seven-letter first word frequency did not differ significantly between lists *F* < 1; nor did six-letter second word frequency, *F* < 1. Imageability values ranged from 258 to 639, with a mean value of 489.32 (SD = 91.18) for seven-letter words and 510.96 (SD = 90.33) for six-letter words. Mean first word imageability was matched across lists, *F*(15,176) = 1.03, *p* = 0.43, *η*_*p*_^2^ = 0.08, as was second word imageability, *F*(15,176) = 1.49, *p* = 0.11, *η*_*p*_^2^ = 0.11.

The construction and presentation of lists were identical to the experiment reported by Cohn et al. [19]. The 192 words were put into pairs and grouped as 12-item lists (16 × 12 = 192). Each list is then rotated such that they are all studied at some stage and act as an intact, rearranged, half-old or new list in the pair and associative recognition tasks. Thus, the 16 lists were rotated to create eight versions of the experiment which were counterbalanced across participants, as were the two different test types (pair and associative recognition task, explained below). Participants studied 120 word pairs (10 lists), as well as three buffer pairs at the beginning and end of presentation. At test they viewed four different types of word pairs: 24 were* intact* pairs, consisting of the old studied pairs; 24 were* rearranged* pairs, derived by randomising the second word of each pair to couple it with a new first word; 24 were* half-old* pairs, consisting of the first word from 12 old studied pairs being joined with 12 new second words and 12 old studied second words being paired with 12 new first words; the final 24 pairs were* new pairs*, consisting of completely new words. Therefore, participants viewed 96 critical test pairs, which were presented in a randomised order (further description of material presentation is provided in Table S1 in Supplemental Materials). Word pairs were written in Times New Roman font and approximately 2 cm in height. E-prime software was used for stimuli presentation and data collection.

### 2.3. Procedure

Participants were all tested individually in a quiet room. During the study phase, they were instructed that they were about to be presented with a large number of word pairs and given 5 s to study each, before having to generate orally a sentence using the two words. They were instructed that there were two rules they should try and follow when creating each sentence. Firstly, they must always use the two words in the order that they appeared; secondly, they should try their best to use the word in the form it appeared in. The experimenter always provided the same example; “So, if one of the words was ‘bank', you should avoid using words such as ‘banked' or ‘banking.'” However, participants were told that if they could only think of a sentence using an alternative ending then they should still provide this as an answer, as the aim of the sentence generation was to facilitate encoding. In this respect, the study procedure was slightly different to that used by Cohn et al., who required the use of the identical form of the words at all times. Each participant completed two practice sentences and the experimenter clarified understanding of the procedure. Participants were shown each word pair for 5 s, with a fixation cross appearing immediately after this had elapsed. Participants were free to begin generating a sentence whilst the words were onscreen if they wished; otherwise this was done whilst the fixation cross was visible. The experimenter keyed a response to indicate whether the participant had successfully generated a sentence. If a reasonable delay had elapsed indicating difficulty with the sentence or if the participant stated that they could not make a sentence, a key was pressed to move onto the next pair. Thus, the fixation cross always remained until the experimenter made a key-press. One-way ANOVA revealed that the mean proportion of pairs successfully formed into sentences did not differ between patients (M = 0.81, SD = 0.15) and controls (M = 0.80, SD = 0.15); *F* < 1. The difference in average length of study phase trended toward significance between patients (M = 25.36 mins, SD = 7.90) and controls (M = 21.00 mins, SD = 5.51); *F*(1, 38) = 3.92, *p* = 0.06, *η*_*p*_^2^ = 0.09. In Cohn et al.'s original article, there were more pronounced differences in encoding time between patients and controls.

The test phase followed immediately after encoding and participants were instructed that they were about to be tested for the word pairs in two different ways. Participants were given the pair and associative identification recognition tests in a counterbalanced order. Examples of both tests were explained using the practice items from the study phase. In the pair recognition task, they were told that they were to respond with “Yes” to pairs of words that contained any two study items (old and rearranged pairs), regardless of whether they were paired together originally. Alternatively, they were told to respond with “No” whenever a pair was comprised of at least one new word (new and half-old pairs). In the associative identification test, participants were told to only respond with “Yes” when the two words on screen formed the original studied pairing (old pairs) and respond with “No” to any other pair (half-old, rearranged, and new pairs). Yes/No responses were recorded using the “v” and “m” keys, with the participant choosing the most comfortable way of depressing these. The keys were counterbalanced across participants, however.

The novel addition to this paradigm was to ask participants how confident they were in their given answer. Therefore, during the instructions, participants were told that following their Yes/No decision, a screen would appear asking them “how confident are you that your answer is correct?” Confidence responses were made on a six-point scale of 0, 20, 40, 60, 80, and 100%. Keys d–k were used for these, with d being 0% and k 100%.

## 3. Results

The first strand of our analysis aimed to compare objective memory performance between patients and controls in the same fashion as Cohn et al.'s [19] study. However, our main focus was to assess the distribution of confidence assigned to responses. This was in order to evaluate the extent to which any identifiable associative recognition impairment in TLE could be accounted for by a memory, or metacognitive account.

### 3.1. Recognition Performance

The proportion of “Yes” responses to the four pair types (new, half-old, rearranged, and intact) for the pair and associative identification tasks are presented in Table 3.

In the pair task, “Yes” responses to rearranged pairs represent hits; in the associative identification task they represent FPs. Signal detection theory (*d*′ prime) was used to calculate the item memory, associative reinstatement, and associative identification measures with FPs of 0 corrected to 0.02 and hit rates of 1 corrected to 0.98. Item memory, derived from performance in the pair recognition task, was calculated by subtracting FPs to new pairs from hits to rearranged pairs. Thus, item memory is reliant on both recollection and familiarity as this simply measures the extent to which participants successfully discriminate rearranged old from new pairings. Associative reinstatement provides a measure of the extent to which a participant gains from items being presented, or reinstated, in their original context (i.e., intact) compared to a different (rearranged) context. This was calculated for the pair task by subtracting the item memory *d*′-score from *d*′-score derived from the proportion of old responses to intact and new pairs. Associative identification describes the extent to which a participant can discriminate between novel and previously studied combinations of word pairs; this was calculated by subtracting FPs to rearranged pairs from hits to intact pairs in the associative identification task. Estimates of recollection and familiarity were computed using a variant of the PDP [22, 44] following Cohn et al. ([19] see pp. 2991-2992 for full rationale and description).

**Figure 1:**
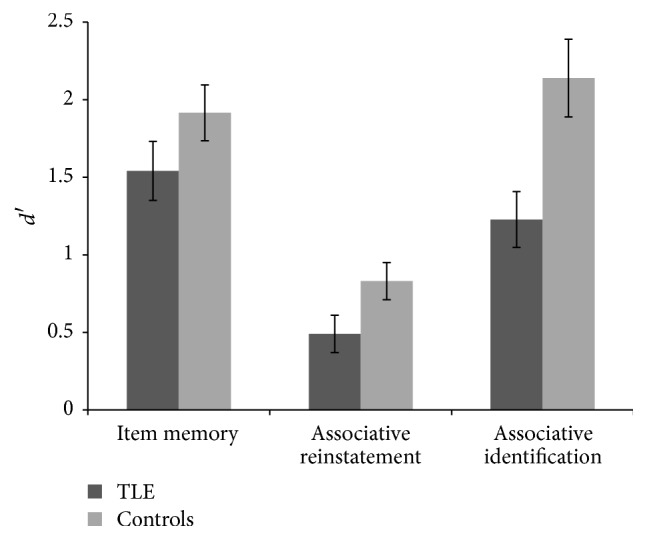
Mean item memory, associative reinstatement, and associative identification *d*′ scores for TLE patients and controls (error bars represent standard error of the mean).

As Figure 1 shows, the TLE group as a whole scored lower than controls on all of the above measures. This difference was, however, not reliable for item memory, *F*(1, 38) = 2.04, *p* = 0.16, and *η*_*p*_^2^ = 0.05, but differed significantly for associative identification, *F*(1, 38) = 9.33, *p* = 0.004, and *η*_*p*_^2^ = 0.20. There was a marginal difference between groups for the associative reinstatement measure, *F*(1, 38) = 3.90, *p* = 0.055, and *η*_*p*_^2^ = 0.09, although notably with a small effect.

The recollection estimate for TLE patients (M = 0.26, SD = 0.24) was numerically lower than controls (M = 0.41, SD = 0.27), but the difference was nonsignificant, *F*(1, 38) = 3.27, *p* = 0.08, and *η*_*p*_^2^ = 0.08. Moreover, the item familiarity estimate was comparable between patients (mean = 1.23, SD = 0.56) and controls (mean = 1.07, SD = 0.68), *F* < 1. To assess relational binding further, hit rates to intact pairs (recall-to-accept) and FPs to rearranged pairs (recall-to-reject) were analysed on the associative identification task, as in Table 3. The TLE group as a whole made a significantly greater number of FPs to rearranged pairs compared to controls, *F*(1, 38) = 11.47, *p* = 0.002, and *η*_*p*_^2^ = 0.23, but did not differ in hit rates to intact pairs, *F* < 1. Therefore, our data are consistent with those of Cohn et al. [19], who similarly displayed that TLE patients appear to be able to successfully utilise recall-to-accept retrieval strategies but have an impaired ability to recall-to-reject.

In summary, TLE patients did not display difficulty in single item recognition, but there was evidence of impairment on measures assessing relational binding and strategic retrieval. Cohn et al. [19] found similar impairments in their TLE group, as well as recollection impairments, but, notably, their sample comprised all postoperative patients and these arguably had more pervasive hippocampal pathology. Cohn et al. also only found item memory impairments in their LTLE group; we ran a laterality analysis and found that our LTLE group has numerically the lowest item memory scores, but a Kruskal-Wallis test revealed no significant difference between groups, *H*(3) = 3.06 and *p* = 0.38. Notably, there were only approximately half the number of patients in our LTLE group as compared to the language dominant LTLE group in Cohn et al.'s study (see Table S3 in Supplementary Materials for laterality subgroup recognition memory performance). Despite this minor inconsistency, due to differences in samples, our results overall provide further evidence for the role of the MTL in relational binding, most notably on the FPs to rearranged pairs, which are the stimuli most pertinent to our examination of metacognition in the recollect-to-reject strategy.

### 3.2. Confidence

The novel contribution of the present experiment was to measure subjective confidence associated with the different response types across the pair and associative recognition tasks. We analysed the extent to which participants' confidence in responses was* sensitive* to item types of varying difficulty, as well as the extent to which confidence changed as a function of correct and incorrect recognition (metacognitive* accuracy*). Confidence levels for each pair type in the pair recognition and associative identification tasks are presented in Table 4.

#### 3.2.1. Sensitivity

We were first interested in whether confidence judgements were sensitive to the difficulty of the different pair types across the two tasks. Confidence judgments across all items, both correct and incorrect, were entered into a 2 (group) × 4 (pair type) × 2 (task) repeated measures ANOVA with group as a between-subjects factor and task and pair type as within-subjects factors. As we predicted, confidence was reliably higher overall in the control group, *F*(1, 38) = 6.28, *p* = 0.02, and *η*_*p*_^2^ = 0.14, and there was a main effect of pair type, *F*(3, 114) = 13.28, *p* = 0.001, and *η*_*p*_^2^ = 0.26, with Bonferroni comparisons confirming that this was in the expected direction with intact pairs giving rise to higher confidence (*p*s < 0.02). There was no interaction between group and pair type, *F* < 1. There was no main effect of task, *F* < 1; because the instructions were identical for the two tasks for three of the pair types, this is not surprising. However, pair type did significantly interact with task, *F*(3, 114) = 5.39, *p* = 0.002, and *η*_*p*_^2^ = 0.12. The means suggest that this is a result of confidence being higher for new and half-old pairs in the associative identification task. Given that the associative identification task only requires an “old” response when the initial bound relationship is retrieved between two words, it is unsurprising that confidence is higher for half-old pairs in particular. There was no interaction between group and task, *F* < 1, or between group, pair type, and task, *F*(3, 114) = 1.73, *p* = 0.17, and *η*_*p*_^2^ = 0.04. The ANOVA shows that the TLE group is significantly less confident overall compared to controls. The fact that there are no significant interactions with group suggests that patients respond no differently in their judgements for the different tasks and pair types than controls. In short, their judgements are sensitive to the difficulty of the task they have been presented and, moreover, the group effect suggests that they are sensitive to their own memory difficulties.

#### 3.2.2. Metacognitive Accuracy

**Figure 2:**
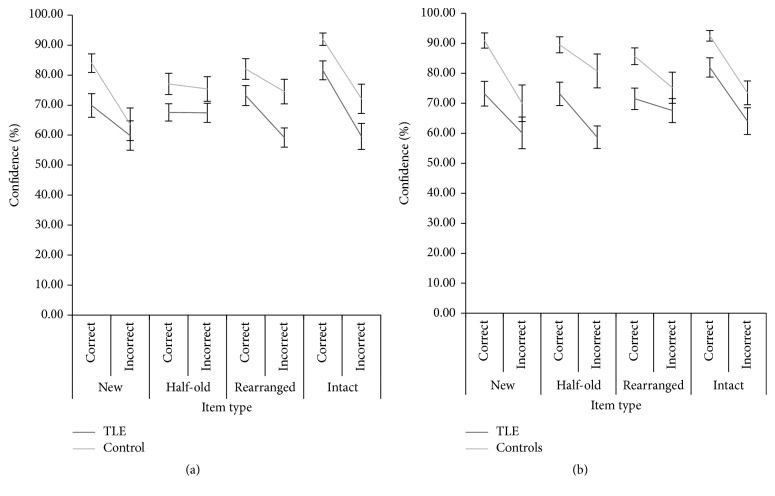
Mean confidence levels for correct and incorrect answers: pair recognition (a) and associative identification (b) tasks.

Metacognitive accuracy refers to a participant's ability to adjust their confidence levels according to whether they answered correctly or not. (Metacognitive accuracy is often measured by nonparametric correlations between confidence judgments and performance. In this sample, there were insufficient data points to calculate gamma.) For metacognitive accuracy, we find significantly higher confidence for correct answers compared to incorrect answers.  Figure 2 shows the mean confidence levels for correct and incorrect answers across the associative recognition task by pair type.

In both tasks, the terms “correct” and “incorrect” for new and half-old pairs reflect correct rejections and FPs, respectively. However, for rearranged pairs in the pair task, “incorrect” refers to items that were not recognised, and in the associative identification task this refers to pairs that were incorrectly endorsed as being intact (hence, “correct” constitutes a correct rejection of the rearranged pair). For intact pairs in both tasks, “correct” refers to hits and “incorrect” refers to misses (“status” in the ANOVA below).

Figure 2 shows that patients and controls display a clear pattern of higher confidence to correct as compared to incorrect answers in both tasks. We compared the pair types for calculating the recognition scores as in Figure 1 (the rearranged and intact pairings), in an ANOVA. (A 2 (group) × 2 (task) × 4 (pair type) × 2 (response type) repeated measures ANOVA was conducted, with group as a between-subjects factor and task, pair type, and response type as within-subjects factors. However, due to a number of participants not making any FPs on certain pair types, this left a data set with only one control participant and 13 TLE patients. Even so, in our resultant analysis that includes 11 control participants, it should be noted that these represent the controls with the highest levels of false positives. Controls (and patients) who did not make false positives for the critical items cannot be included in this analysis.) For this, there were 20 TLE patients and 11 controls in a 2 (group) × 2 (task) × 2 (pair type) × 2 (status) design. In this analysis, the group effect was reduced and was no longer significant, *F*(1, 29) = 3.00, *p* = 0.09, and *η*_*p*_^2^ = 0.09. As expected, however, there was a highly significant main effect of response type, *F*(1, 29) = 36.66, *p* = 0.001, and *η*_*p*_^2^ = 0.56, such that correct responses were assigned higher confidence than incorrect responses. This suggests that even the worse performing participants on both tasks are metacognitively competent; they are able to accurately assign higher confidence to correct answers and significantly shift their confidence downward to incorrect answers. There was no effect of task, *F*(1, 29) = 2.64, *p* = 0.12, and *η*_*p*_^2^ = 0.08, indicating that the different instructions did not influence the way confidence was assigned to these critical pair types. There was a marginal effect of pair type in this analysis, *F*(1, 29) = 3.96, *p* = 0.06, and *η*_*p*_^2^ = 0.12, again suggesting that overall confidence was higher for intact pairs than rearranged pairs. No interactions were found between the within-subject factors and group and, of the within-subject factors, only status and pair type interacted significantly, *F*(1,29) = 13.83, *p* = 0.001, and *η*_*p*_^2^ = 0.32, with the means showing that there is a greater difference between correct and incorrect judgements for the intact compared to rearranged pairs.

## 4. Discussion

The experiment sets out with the aim of further examining the potential role of metacognitive impairments in TLE following inconsistent findings in the literature. Specifically, we examined whether a metacognitive failure might be found in the associative identification task which characterises the recollection deficit in TLE [19]. To this end, we used a procedure whereby postrecognition confidence judgements were reported for each pair by participants. Although we found no significant impairment in PDP derived recollection estimates in the TLE participants, our study has replicated one of the key associative deficits observed by Cohn et al. - an inability to retrieve associative binding information at test to differentiate intact from recombined pairs of previously studied items in an associative identification task (i.e., recall-to-reject). As well as replicating this deficit, critically, the confidence data suggest that failure in this aspect of the task is not driven by faulty metacognitive monitoring and lack of subsequent strategic control of recollection. Before we interpret these findings in light of the confidence data, it is worth exercising a little caution since some of our key analyses, whilst significant, were based on a small sub sample of the controls, who whilst performing better than the patient group, may not have been fully representative of the general population.

The recent study by Fleming et al. [31] illustrated intact metacognition for simple Yes/No recognition in surgically resected TLE patients using confidence judgments. Our data expand upon these findings and provide an important demonstration of how confidence data can help us understand the basis of associative recognition deficits in TLE. We found that in terms of sensitivity and accuracy measures of metacognition, the TLE group were aware both of which pairs were (objectively) more difficult and also made confidence judgements which were reflective of their performance. Interestingly, the level of confidence in the TLE group was the highest for correct responses for intact pairs. Thus, we might understand better the failure to use a recall-to-reject strategy: patients with TLE have the subjective impression that recombined pairs are more difficult and are less confident in their responses for such items. Indeed, in general, the TLE participants report that they are less confident, in contrast with the first studies of metacognition in this group, which found overconfidence [27, 28]. An ideal test of the metacognitive competencies of people with TLE is to examine their awareness on the very tasks which illustrate best their memory deficit, as we have done here.

The confidence findings are of interest since they reveal that, as Cohn et al. [19] suggest, there is not a strategic failure involved in the associative identification error, but a lower level deficit in the binding of representations from a prior study phase. We interpret our results as suggesting that TLE patients detect the difficulty of the task; they then fail to retrieve information to reconcile the higher level of familiarity and thus report a lower level of confidence. The direct measurement of confidence shows that the FP errors in TLE are not born of overconfidence but that when prompted to use a recollect-to-reject, the TLE groups are, indeed, less certain in their performance.

Because we found evidence suggesting a specific deficit in recollection coupled with a corresponding preservation of metacognitive awareness, our findings are incompatible with a “misrecollection” account of false positives in TLE, which has been shown in older adults' [45] and Alzheimer's patients' [46] high confidence false positive errors, whereby presumably strategic factors are at play. However, Gallo et al. [47] recently found patients with mild Alzheimer's disease to show a similar preservation in the confidence-accuracy relationship in light of recollection impairments. Therefore, evidence accumulated so far strongly suggests that recollection impairments in the context of MTL damage do not necessarily cause a breakdown in the accurate subjective feeling of confidence.

Neuroscientific accounts of metacognition tend to place the emphasis on the frontal lobes [48]; therefore our findings can be incorporated into a model of metacognition whereby a frontal network interacts with the temporal lobe memory system (see [19, 49] for further discussion of such a system). Given that recent studies, including ours, have now examined a variety of different types of metacognitive judgment under different memory test conditions with a range of TLE patients and found no evidence for impairments, it may be that metacognitive monitoring abilities are only likely to be impaired in this group following secondary damage to frontal areas. Although not found in the current sample, or that of Howard and colleagues' [12, 29, 30], discrete impairments in frontally mediated executive functions and working memory do occur in TLE and are currently believed to result from either long-term seizure propagation to the frontal lobes or the detrimental effects on executive regions from antiepileptic medications [11, 50]. Thus, it may be that a specific subgroup of TLE patients with more extensive cross-domain neuropsychological impairments show impaired metacognitive abilities.

Material specificity is one of the cornerstones of neuropsychological investigation with this clinical group and we acknowledge that a limitation of the present study is the heterogeneous nature of the patient sample. With this group, though, we were able to decompose a robust memory paradigm to better understand a particular deficit on a specific task. It is still important to bear in mind differences that may arise in patients with left and right epileptic foci for verbal and visual material, respectively. A number of behavioural measures of recognition memory vary according to hemisphere of localisation such as accuracy and response bias [51], but it is unclear in TLE the extent to which metacognitive function is affected differently between each cerebral hemisphere. Further work in this area with specific subgroups of patients is thus likely to both add to our understanding of awareness of deficit in TLE and contribute to neuroscientific accounts of metacognition.

## 5. Conclusions

Thus, we find that our TLE group are able to use item memory and familiarity relatively well but have a particular deficit in detecting when previously studied items have been recombined into a novel combination. Insofar as one must be aware of the operations of the memory system and the difficulty posed by certain memory decisions, we do not find a strategic deficit in our TLE group. They are at least appropriately lacking in confidence, in that they are less confident than controls in general, but when the task is more difficult they have proportionately lower confidence. In general, we suggest that clinicians and researchers may find it helpful to adopt a metacognitive viewpoint as we have done here: much can be learned by operationalising and assessing patient's subjective reports of their experiences in this manner.

## Supplementary Material

Supplementary Materials for this article include additional information regarding clinical characteristics of the epilepsy sample; a detailed outline of the experimental design; and a breakdown of recognition memory performance for the epilepsy group separated by laterality of seizure focus.

## Figures and Tables

**Table 1:** Mean (and standard deviation) demographic characteristics for TLE and controls and TLE characteristics.

	TLE *n* = 22	Controls *n* = 18
Age	41.32 (11.22)	37.72 (13.93)
Gender (female : male)	18 : 4	8 : 10
Education (years)	12.82 (2.26)	13.17 (1.72)
NART (FSIQ)	107.90 (9.95)	109.78 (8.93)
Age of onset	20.75 (15.07)	—
Duration of illness (yrs)	18.86 (14.73)	—
Laterality (left : right : bilateral)	9 : 9 : 4	—
Handedness (right : left)	17 : 5	18 : 0

Note: NART = National Adult Reading Test [33]; TLE = temporal lobe epilepsy.

**Table 2:** Summary of neuropsychological assessment (standard deviation in parentheses).

Test	TLE	Controls	*F* statistic	*p* value
*WASI (index scores)*				
VIQ	105.21 (15.39)	105.72 (14.92)	0.01	0.92
PIQ	102.63 (12.72)	109.72 (13.69)	2.74	0.11
FSIQ	104.76 (13.58)	108.94 (13.65)	0.94	0.34

*BMIPB (z-scores)*				
Figure immediate	−1.35 (1.12)	0.06 (1.18)	14.56	0.001
Figure delayed	−0.88 (−0.88)	0.39 (0.91)	19.64	0.001
List learning	−0.28 (1.38)	0.33 (1.14)	2.20	0.15
Postinterference list	−0.61 (1.18)	0.28 (0.73)	7.74	0.01
Story immediate	−1.13 (1.15)	−0.06 (0.77)	11.20	0.002
Story delayed	−1.26 (1.02)	0.11 (0.93)	19.33	0.001

*WRMT (raw scores)*				
Words	44.00 (4.41)	48.22 (1.66)	14.74	0.001
Faces	39.00 (6.13)	43.44 (4.10)	6.90	0.01

*D-KEFS fluency (z-scores)*				
FAS verbal	−0.55 (1.04)	0.13 (0.96)	1.78	0.19
Category	−0.49 (0.98)	0.07 (0.90)	1.97	0.17

Max. digit span forward	7.00 (1.11)	6.94 (1.06)	0.03	0.87
Max. digit span backward	5.23 (1.34)	5.78 (1.17)	1.83	0.19

*HADS (raw scores)*				
Anxiety	8.59 (3.88)	5.83 (3.47)	5.52	0.02
Depression	4.80 (2.98)	3.56 (2.81)	1.82	0.19

Note: TLE = temporal lobe epilepsy, NART = National Adult Reading Test [33], WASI = Wechsler Abbreviated Scale of Intelligence [34], VIQ = verbal IQ, PIQ = performance IQ, FSIQ = full scale IQ, BMIPB = BIRT Memory and Information Processing Battery [35], WRMT = Warrington Recognition Memory Test [36], D-KEFS = Delis-Kaplan Executive Function Scale [37], and HADS = hospital anxiety and depression rating scale [38].

**Table 3:** Mean (SD) proportions and standard deviations of “Yes” responses to each pair type in the pair and associative identification tasks.

Group	Recognition responses			
	New	Half-old	Rearranged	Intact
*Pair task*				
Control	0.07 (0.09)	0.23 (0.15)	0.60 (0.20)	0.82 (0.18)
TLE	0.15 (0.18)	0.40 (0.19)	0.60 (0.19)	0.75 (0.21)

*AI task*				
Control	0.01 (0.02)	0.06 (0.10)	0.14 (0.12)	0.76 (0.19)
TLE	0.11 (0.21)	0.17 (0.22)	0.33 (0.21)	0.73 (0.19)

Note: TLE = temporal lobe epilepsy; AI = associative identification.

**Table 4:** Mean (SD) percentage confidence assigned to answers for each pair type overall in the pair and associative identification tasks.

Group	Metacognitive sensitivity			
	New	Half-old	Rearranged	Intact
*Pair task*				
Control				
M (SD)	83.01 (13.31)	77.04 (15.03)	80.79 (13.97)	88.66 (9.47)
TLE				
M (SD)	70.76 (18.58)	70.22 (14.31)	71.21 (14.80)	79.97 (14.13)

*AI task*				
Control				
M (SD)	85.83 (16.77)	85.93 (15.10)	81.90 (13.93)	86.34 (11.37)
TLE				
M (SD)	74.17 (19.31)	73.37 (18.57)	73.26 (17.30)	79.55 (14.90)

Note: TLE = temporal lobe epilepsy; AI = associative identification.
