# Supplementary material for: Assessing a Metacognitive Account of Associative Memory Impairments in Temporal Lobe Epilepsy
Source: Epilepsy Res Treat. 2016 Sep 19;2016:6746938. doi: 10.1155/2016/6746938 (PMC5046021; doi:10.1155/2016/6746938)
Supplement: Supplementary file 1 — Supplementary Materials for this article include additional information regarding clinical characteristics of the epilepsy sample; a detailed outline of the experimental design; and a breakdown of recognition memory performance for the epilepsy group separated by laterality of seizure focus. [file 6746938.f1.pdf]

Table S1. Summary of clinical characteristics in temporal lobe epilepsy group.

Patient	Age	Gender	Diagnosis	Evidence	Lesion status	Onset (yrs)	Duration (yrs)
1	55	F	RTLE	MRI/V-EEG/Sem/NPA	MTL DNET	52	3
2	54	M	RTLE	MRI/EEG/Sem/NPA	Resection	16	38
3	55	F	LTLE	MRI	Resection	18 mnths	46
4	33	M	LTLE	MRI	MTL DNET	16	17
5	30	M	LTLE	MRI/EEG/Sem	Left lesion	17	13
6	26	F	RTLE	EEG/V-EEG/Sem	Unknown	17	9
7	48	F	LTLE	MRI	MT sclerosis	5	43
8	38	F	BTLE	EEG/MRI/CT all normal	Unknown	14	24
9	42	F	LTLE	EEG/Sem, MRI normal	Unknown	4	38
10	46	F	RTLE	EEG/Sem/MRI	Probable right lesion	44	2
11	56	F	RTLE	MRI	Right hippocampectomy	23	22
12	47	F	LTLE	MRI	Left HS	14	33
13	38	F	BTLE	MRI	Right posterior H atrophy; Left anterior H atrophy	0	38
14	39	F	LTLE	EEG/Sem	Unknown	21	18
15	18	F	RTLE	EEG/Sem	Unknown	14	4
16	49	M	RTLE	CT/Sem	RTL damage secondary to HSE	25	5
17	38	F	LTLE	MRI	Resected left posterior temporal cavernous angioma	23	15
18	43	F	LTLE	MRI	Possible LGA in left temporal horn	40	3
19	44	F	RTLE	MRI	Right resection following benign meningioma	33	11
20	57	F	BTLE	MRI/EEG normal	Unknown	51	6
21	23	F	RTLE	EEG/Sem	Unknown	21	2
22	30	F	BTLE	EEG/Sem	Unknown	5	25

Note: TLE = temporal lobe epilepsy, RTLE = right temporal lobe epilepsy, LTLE = left temporal lobe epilepsy, BTLE = bilateral temporal lobe epilepsy, MRI = magnetic resonance imaging, V-EEG = video-electroencephalography, Sem = semiology, NPA = neuropsychological assessment, IC-EEG = intracranial electroencephalography, CT = computed tomography, MTL = medial temporal lobe, DNET = dysembryoplastic neuroepithelial tumour, H = hippocampus, HS = hippocampal sclerosis, HSE = herpes simplex encephalitis, LGA = low grade astrocytoma.

Explanation of experimental design and material presentation.

For the benefit of the reader we provide Table S2 below which details how each of the 16 lists of 12 word pairs is rotated and what exactly is 'done' with the words. Essentially, in each of the eight versions of the task two lists are rotated to the top to become a) a study list or not; b) one of the four different pair types at test; and c) appear in either the pair or associative identification task.

When a list was designated as 'rearranged', the first seven letter words remained and for each list we simply randomised the second word of the pair; this remained constant for each participant (so each list was not randomised to create new rearranged pairs for every person). Items were of course randomised within the test phase, however. For half-old pairs, both of the words that were studied in a pair designated as 'half-old' appeared again in each test, except the first and second words were re-paired with a different word. So in Table 1 below, the reader will see that for each test (pair or AI) one list of words was both studied and tested when designated 'half-old' – splitting the list up as just described allows for the creation of 24 items for participants to be tested on.

Table S2. Construction of materials in associative recognition paradigm

Participant 1

List	*Study?*	*Item type (for test)*	*Test*
1	Study	Intact	Pair
2	Study	Intact	Pair
3	Study	Intact	AI
4	Study	Intact	AI
5	Study	Rearranged	Pair
6	Study	Rearranged	Pair
7	Study	Rearranged	AI
8	Study	Rearranged	AI
9	Study	Half old	Pair
10	Study	Half old	AI
11		Half old	Pair

12		Half old	AI
13		New	Pair
14		New	Pair
15		New	AI
16		New	AI

Participant 2

List	*Study?*	*Item type (for test)*	*Test*
3	Study	Intact	Pair
4	Study	Intact	Pair
5	Study	Intact	AI
6	Study	Intact	AI
7	Study	Rearranged	Pair
8	Study	Rearranged	Pair
9	Study	Rearranged	AI
10	Study	Rearranged	AI
11	Study	Half old	Pair
12	Study	Half old	AI
13		Half old	Pair
14		Half old	AI
15		New	Pair
16		New	Pair
1		New	AI
2		New	AI

Participant 3

List	*Study?*	*Item type (for test)*	*Test*
5	Study	Intact	Pair
6	Study	Intact	Pair
7	Study	Intact	AI

8	Study	Intact	AI
9	Study	Rearranged	Pair
10	Study	Rearranged	Pair
11	Study	Rearranged	AI
12	Study	Rearranged	AI
13	Study	Half old	Pair
14	Study	Half old	AI
15		Half old	Pair
16		Half old	AI
1		New	Pair
2		New	Pair
3		New	AI
4		New	AI

Table S3. Recognition performance for controls and TLE seizure laterality groups.

	Recognition measures					
Participants	Proportion of rearranged FPs (Associative Identification task)	Associative Reinstatement (*d'*)	Item memory (*d'*)	Associative Identification (*d'*)	R	F(*d'*)
Control mean	0.14	0.83	1.92	2.14	0.41	1.07
SD	0.13	0.49	0.77	1.06	0.27	0.68
LTLE						
1	0.42	0.80	1.76	1.16	0.27	1.56
2	0.25	0.90	0.71	0.78	0.00	0.71
3	0.21	0.54	1.95	0.81	0.25	1.47
4	0.67	0.47	0.89	0.51	0.07	0.79
5	0.29	0.20	1.03	0.65	0.00	1.08
6	0.50	-1.19	-1.01	-0.31	0.22	0.00
7	0.58	0.62	2.08	0.75	0.04	2.00
8	0.08	0.52	2.49	3.16	0.58	1.21
9	0.21	0.23	2.13	1.98	0.60	1.45
10	0.5	1.62	1.08	0.81	0.00	1.63
Mean	0.37	0.47	1.31	1.03	0.20	1.19
SD	0.19	0.71	1.02	0.94	0.23	0.57
RTLE						
1	0.79	0.55	1.30	0.00	0.00	1.55
2	0.08	-0.22	1.11	0.73	0.38	0.62
3	0.13	0.74	2.19	1.8	0.48	1.36
4	0.25	-0.01	2.50	1.85	0.6	2.06
5	0.17	0.85	2.61	2.13	0.54	1.70
6	0.42	0.30	0.71	1.16	0.04	0.65
7	0.63	1.55	0.88	0.84	0	1.30
8	0.46	0.15	1.93	1.28	0.27	1.71
Mean	0.37	0.49	1.65	1.22	0.29	1.37
SD	0.25	0.56	0.75	0.70	0.25	0.51
BTLE						
1	0.17	0.73	2.73	2.13	0.63	1.60
2	0.29	0.37	2.19	0.89	0.45	1.46
3	.00	0.25	1.50	2.49	0.28	0.03
4	0.17	0.80	1.13	1.39	0.04	1.09
Mean	0.16	0.54	1.89	1.73	0.35	1.05
SD	0.12	0.27	0.71	0.72	0.25	0.71

Note: TLE = temporal lobe epilepsy; LTLE = left temporal lobe epilepsy; RTLE = right temporal lobe epilepsy; BTLE = bilateral temporal lobe epilepsy; FPs = false positives.
